# Photobiomodulation as an Alternative Approach for Bell′s Palsy: Case Reports

**DOI:** 10.1155/crid/2340155

**Published:** 2026-10-14

**Authors:** Omar Andrés Zamorano Castro, María Cecilia Aveiga-Zambrano, Gilmar Hugo Paredes-Muñoz, Luis Fernández Cotera, Williams Ronald Olivera-Acuña, Debora Brasilina Duarte, Ángel Terrero-Pérez, Renato Yassutaka Faria Yaedú, Mariela Peralta-Mamani

**Affiliations:** ^1^ Faculdade do Centro Oeste Paulista Bauru São Paulo Brazil; ^2^ Universidad Católica de Santa Maria Arequipa Peru; ^3^ Private Practice Orlando Florida USA; ^4^ Integrated Clinic Universidad Peruana Los Andes Huancayo Peru; ^5^ Bauru School of Dentistry University of São Paulo Bauru São Paulo Brazil usp.br; ^6^ Hospital for Rehabilitation of Craniofacial Anomalies University of São Paulo Bauru São Paulo Brazil usp.br

**Keywords:** Bell’s palsy, case report, facial paralysis, low-level light therapy

## Abstract

**Background:**

Bell′s palsy (BP), also known as idiopathic facial paralysis, is an idiopathic peripheral neuropathy that unilaterally affects the facial musculature. Its management includes various therapeutic modalities, among which photobiomodulation (PBM) has shown positive effects on neuromuscular regeneration.

**Objective:**

To describe the clinical outcomes of PBM in two patients with BP, one chronic and one acute.

**Methods:**

The first patient, a 58‐year‐old woman, presented with left‐sided BP of 4 years′ duration and had undergone multiple unsuccessful treatments. The second patient, a 15‐year‐old adolescent, was diagnosed 2 weeks prior to the initiation of PBM and underwent pharmacological therapy for 2 weeks, facial physiotherapy five times per week for 5 months, and auriculotherapy once weekly for 3 months. Both patients received infrared laser therapy (808 nm, 3 J/point) following individualized, progressive protocols.

**Results:**

In the chronic case, there was a significant reduction in orofacial pain from the first session and gradual improvement in facial mobility up to the fifteenth session, with follow‐up extending to 18 months. Although complete facial symmetry was not restored, the patient reported enhanced self‐confidence and quality of life. In the acute case, complete pain relief was achieved within a few sessions, and full functional recovery was observed after 14 sessions, with restoration of facial symmetry and emotional well‐being. Clinical outcomes remained stable at the three‐year follow‐up.

**Conclusions:**

PBM proved to be an effective tool for managing both chronic and acute BP, promoting analgesia, motor recovery, and general well‐being. Its integration into multidisciplinary treatment protocols may optimize therapeutic outcomes.

## 1. Introduction

Bell′s palsy (BP), also known as idiopathic facial paralysis, was first described in the 19th century by the Scottish anatomist Charles Bell [1]. It is a peripheral neuropathy characterized primarily by motor dysfunction resulting from impairment of the facial nerve (cranial nerve VII). This nerve originates at the pontomedullary junction and traverses the temporal bone, giving rise to branches that innervate the tongue and facial muscles. Its function is essential for facial mobility and expression, as well as for muscular activity within the stomatognathic system [2]. The incidence of BP is approximately 30 cases per 100,000 individuals, with no sex predilection. However, age is considered an important prognostic factor, as older patients are less likely to achieve complete functional recovery. Diabetes mellitus (DM) is another recognized risk factor, with delayed treatment being associated with lower recovery rates. Patients with DM who begin treatment later in the course of the disease have recovery rates of approximately 70%–75%, whereas early treatment may increase recovery to approximately 82% [3].

The pathophysiology of BP is believed to involve inflammation and edema of the facial nerve, particularly within the facial canal near the stylomastoid foramen, leading to impaired motor impulse transmission to the facial musculature [4]. Viral infections, transient ischemia, and systemic comorbidities such as arterial hypertension have also been implicated [1, 4].

Clinically, BP typically presents as an acute unilateral peripheral facial paralysis characterized by facial asymmetry, flattening of the nasolabial fold, eyebrow ptosis, incomplete eyelid closure, and impaired facial expressions [1, 5]. Dysfunction of the lacrimal and salivary glands may also occur because of facial nerve involvement [4, 6, 7]. Additional manifestations may include altered taste, hyperacusis, and periauricular discomfort resulting from involvement of nonmotor fibers carried by the facial nerve [8].

Although BP is primarily a motor disorder, some patients may present with concomitant facial or cervical pain. These symptoms are not necessarily a direct manifestation of facial nerve dysfunction but may reflect concurrent involvement of the trigeminal pathway or another coexisting orofacial condition. The trigeminal nerve provides the major sensory innervation of the face, and its involvement has been reported in patients with BP presenting with facial pain, neck pain, paresthesia, or sensory deficits. Therefore, BP and orofacial pain should be regarded as distinct, although potentially coexisting, clinical entities, requiring independent clinical evaluation and therapeutic planning [9].

Early treatment significantly influences prognosis and commonly includes corticosteroids, physiotherapy, peripheral nerve stimulation, and photobiomodulation (PBM) [7]. These therapies may act synergistically to optimize facial nerve recovery without therapeutic interference [4].

PBM has demonstrated beneficial effects on peripheral nerve regeneration through PBM of cellular metabolism, reduction of inflammation, and stimulation of tissue repair [10, 11]. In patients presenting with concomitant orofacial pain, PBM may also be directed to painful muscles or sensory regions as an adjunctive approach. Thus, although similar irradiation parameters may be employed, the therapeutic objective differs according to the target tissue, aiming either to support facial nerve recovery or to alleviate the associated painful condition [9].

Importantly, PBM is minimally invasive, well tolerated, and may be safely combined with conventional therapies [7, 12]. Therefore, this study is aimed at presenting two clinical cases of patients diagnosed with BP, one with a 4‐year history and another with a recent 2‐week onset, and reporting the outcomes following PBM treatment.

## 2. Case Reports

### 2.1. Case 1

A 58‐year‐old woman presented to the clinic reporting “pain and loss of sensation on the left side of the face.” She reported having experienced significant stress 4 years prior, after which she developed facial paralysis on the left side, ultimately diagnosed as BP.

Despite undergoing pharmacological treatment, electrotherapy for 2 months, and acupuncture, she reported no meaningful improvement in facial motor function. Her medical history was notable for gastritis.

In addition to the facial motor deficit, the patient complained of persistent spontaneous pain, particularly in the left nasal region. Clinical examination demonstrated two distinct findings. First, signs compatible with chronic BP were observed, including facial asymmetry, flattening of the left nasolabial fold, inferior deviation of the left labial commissure, impaired facial mimicry, and limited facial expressions (Figure 1A,B). Second, musculoskeletal examination revealed bilateral tenderness of the masseter, temporalis, sternocleidomastoid, and medial and lateral pterygoid muscles, consistent with myofascial pain. Pain intensity was assessed using the Visual Analog Scale (VAS), with most trigger points scoring six (VAS = 6). Extraoral examination also revealed a hematoma on the left side of the chin related to a dental extraction performed 7 days earlier.

**Figure 1:**
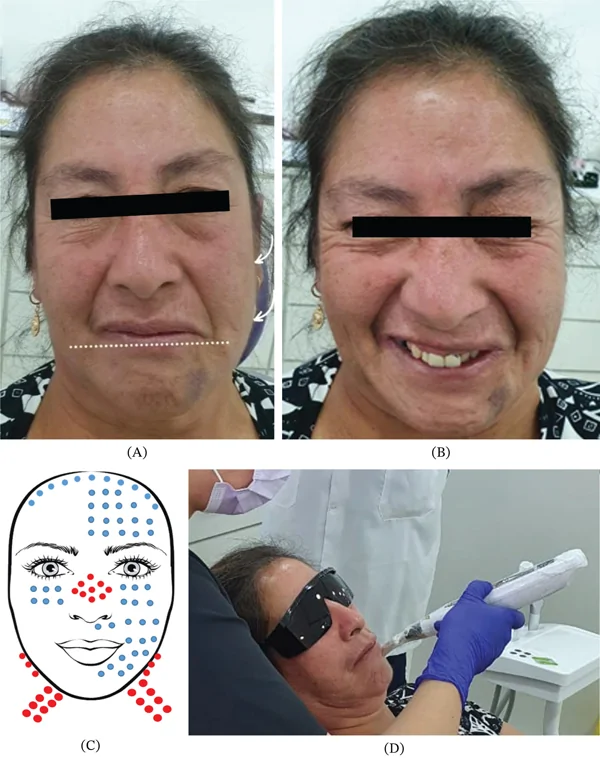
Initial clinical photographs and photobiomodulation (PBM) application protocol in Case 1. (A,B) Initial extraoral photographs showing facial asymmetry and a hematoma on the left side of the chin following dental extraction. (C) Schematic representation of the PBM application points. Blue points indicate irradiation sites for facial motor rehabilitation, whereas red points represent the areas of greatest pain intensity treated as myofascial trigger points. (D) Clinical application of PBM.

PBM was initiated with two complementary therapeutic objectives. Irradiation over the affected left hemiface was performed to promote peripheral facial nerve recovery and improve neuromuscular function associated with BP. The application sites were distributed according to the anatomical regions corresponding to the facial muscles innervated by the facial nerve, aiming to stimulate motor recovery. Additional irradiation was directed to clinically identified myofascial trigger points in the masticatory and cervical muscles, which represented a distinct therapeutic target for pain management.

The Laser Duo device (MMOptics, São Carlos, Brazil), a GaAlAs diode laser operating at 808 nm, 100 mW, continuous mode, was used in contact with the skin. The irradiation parameters were: spot size 0.030 cm^2^, irradiance 3.33 W/cm^2^, fluence 100 J/cm^2^, 30 s per point, 3 J per point, and 1‐cm spacing between irradiation points. During the initial phase, 80 points were irradiated (2400 s per session), twice weekly for 2 weeks (Figure 1C,D). Although identical dosimetric parameters were employed, the biological targets differed according to the treated tissues.

After the first PBM session, the patient reported marked reduction in myofascial pain and improved cervical mobility without discomfort. After four sessions, pain remained only at a limited number of trigger points. Consequently, treatment became predominantly focused on the facial motor deficit, reducing the number of irradiation points to 44 while maintaining the same dosimetric parameters. Sessions continued twice weekly for an additional 4 weeks (1320 s per session; cumulative irradiation time 20,160 s). Pain progressively decreased throughout treatment (VAS 4–5 at the fifth session, 3–4 at the sixth session, 2–3 at the 10th session, and 1–2 at the 12th session). Although complete pain resolution was not achieved, the reduction in myofascial pain was clinically meaningful and positively affected the patient′s quality of life.

Improvement in facial motor function became evident from the eighth session onward. Progressive recovery of facial mobility, improvement of facial symmetry, better definition of the nasolabial fold, improved position of the oral commissure, and enhanced facial expressions were observed (Figure 2A–E). Retrospective assessment using the House–Brackmann Facial Nerve Grading System [13] classified the patient as Grade IV at baseline, improving to Grade II after completion of the PBM protocol. The patient also reported subjective improvement in facial sensation and greater comfort during mandibular movements.

**Figure 2:**
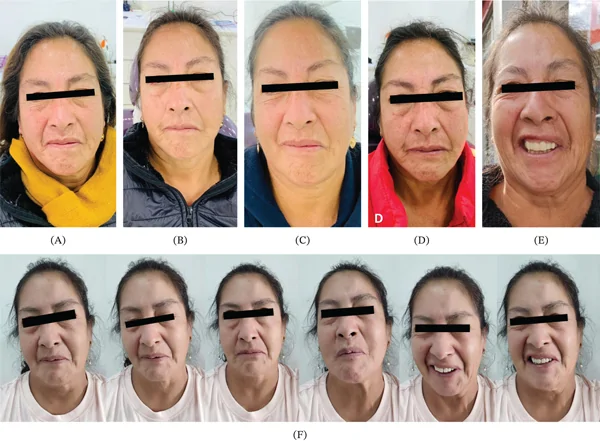
Photographs showing the patient′s clinical evolution during treatment. (A) Third session. (B) Fifth session. (C) Sixth session. (D) Twelfth session. (E) One‐month follow‐up. (F) Extraoral photographs showing different facial expressions at the 18‐month follow‐up.

At the 18‐month follow‐up, facial motor improvements remained stable, with a more harmonious facial appearance and greater ease in smiling (Figure 2F). The patient reported increased self‐confidence, improved social interaction, and better overall quality of life. No adverse effects related to PBM were observed during treatment or follow‐up.

### 2.2. Case 2

A 15‐year‐old black female patient with no history of systemic diseases presented with left‐sided facial paralysis. She had previously been evaluated by a neurologist, who diagnosed BP. At presentation, she had been taking prednisone (20 mg/day) and fluoxetine (20 mg/day) for 2 weeks, with reduced salivary flow reported during corticosteroid therapy.

The patient underwent facial physiotherapy sessions five times per week for five consecutive months, in addition to auriculotherapy once per week for 3 months.

Clinical examination demonstrated two concurrent conditions. First, the patient exhibited characteristic signs of BP, including facial asymmetry during speech, impaired facial expressions, predominant movement on the right side, and marked limitation of movement on the affected left hemiface (Figure 3A). Second, clinical examination of the masticatory and cervical muscles revealed tenderness on palpation of the masseter, temporalis, sternocleidomastoid, and medial and lateral pterygoid muscles. Pain intensity ranged from VAS 4–9 in the masseter and pterygoid muscles and VAS 6–7 in the sternocleidomastoid muscles. The patient also reported facial and cervical pain, difficulty sleeping on the affected side, and leakage of food and liquids through the left oral commissure during mastication.

**Figure 3:**
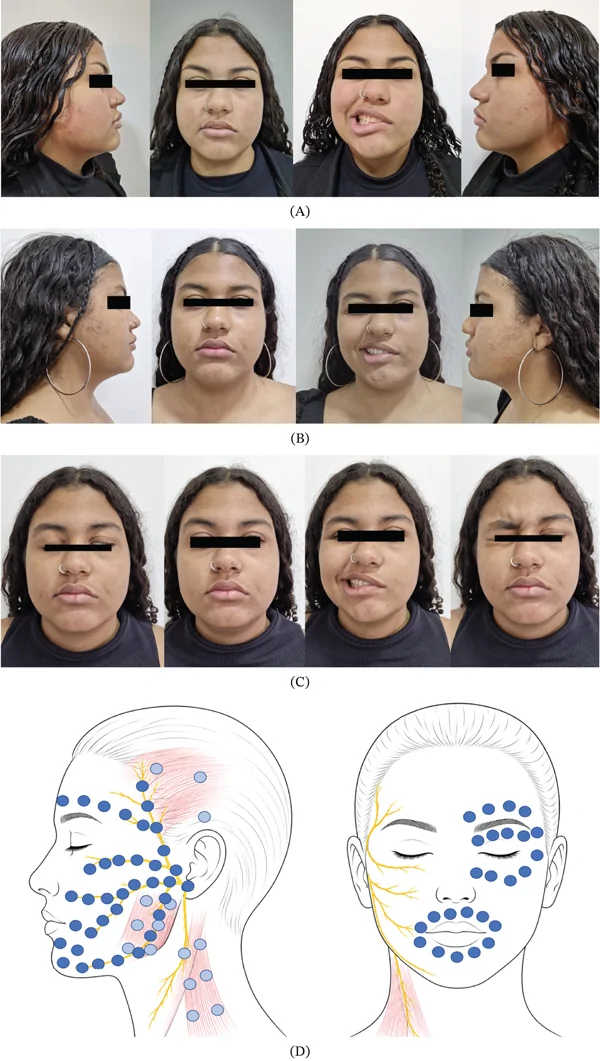
Clinical photographs showing treatment progression and the photobiomodulation (PBM) protocol in Case 2. (A) Initial presentation showing facial asymmetry and restricted movement of the left hemiface. (B) After five sessions, persistent food leakage and ocular dryness requiring lubricating eye drops. (C) At the 11th session, improved facial expression and eyelid closure. (D) PBM application sites: light blue points indicate myofascial trigger points for pain management, while dark blue points represent sites for facial motor rehabilitation.

PBM was initiated with two therapeutic objectives: to promote neuromuscular recovery of the facial nerve and to manage concomitant myofascial pain. Laser irradiation was therefore applied over the affected left hemiface as well as over painful muscular trigger points. For neuromuscular rehabilitation, irradiation followed the anatomical course of the facial nerve, beginning at the stylomastoid foramen and extending over the temporal, zygomatic, buccal, marginal mandibular, and cervical branches (Figure 3D). Separate irradiation was performed over painful trigger points of the masticatory and cervical muscles identified during clinical examination.

Treatment was performed using the DMC Therapy EC laser (GaAlAs diode, 808 nm, 100 mW, continuous wave, contact mode, spot size 0.0984 cm^2^, 3 J per point, 30 s per point, 1‐cm spacing between points). Although identical dosimetric parameters were maintained throughout treatment, the biological targets differed according to the irradiated tissues.

During the first session, 98 irradiation points were treated. Immediately after treatment, the patient reported substantial reduction in muscle pain and improved cervical mobility. Forty‐eight hours later, the second session demonstrated complete pain resolution (VAS = 0) in several trigger points of the masseter and sternocleidomastoid muscles, and 85 irradiation points were performed. At the third session, only two trigger points remained painful in the left medial pterygoid muscle (VAS 4–5), whereas mild discomfort persisted in the right masseter and pterygoid muscles (VAS 3–7). Between the third and sixth sessions, approximately 80 irradiation points were applied, progressively adjusted according to the remaining painful trigger points, and the facial motor deficits.

By the fifth session, the patient still experienced food leakage during mastication and ocular dryness, requiring lubricating eye drops (Figure 3B). Complete resolution of myofascial pain was achieved by the seventh session (VAS = 0). Thereafter, treatment became predominantly directed toward facial motor rehabilitation, with 65 irradiation points concentrated on the affected hemiface, including the orbicularis oris muscle. At the eighth session, masticatory function improved, and food leakage was no longer observed.

Between the eighth and 11th sessions, the number of irradiation points was progressively reduced to 50 according to the remaining motor impairment. Improvement in facial expression and eyelid closure was observed, and eight additional points were applied around the orbicularis oculi muscle (total of 58 points) (Figure 3C). At the 12th session, further recovery of the orbicularis oris and orbicularis oculi muscles was observed. By the 13th session, normal eyelid closure had been restored, ocular lubrication was no longer required, and only 30 irradiation points were necessary. During the 14th session, treatment was restricted to the residual motor limitation of the orbicularis oris muscle.

Two months later, the patient remained free of myofascial pain and demonstrated marked recovery of facial motor function (Figure 4A). Mild residual limitation of the orbicularis oris muscle persisted, and 12 additional irradiation points were applied to this region during the final treatment sessions (Figure 4B). At the 2‐month and 6‐month follow‐ups (Figure 4C,D), the patient exhibited normal facial expressions, absence of muscle pain, complete functional recovery. Retrospective assessment using the House–Brackmann Facial Nerve Grading System [13] demonstrated recovery from Grade IV at baseline to Grade I (normal facial function) after completion of the PBM protocol.

**Figure 4:**
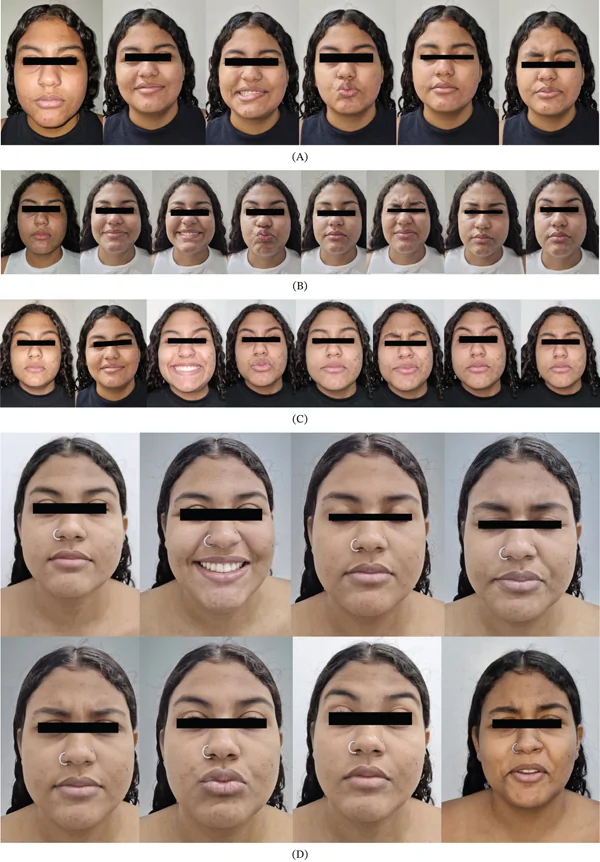
(A,B) Extraoral photographs at the 13th and 14th sessions. (C,D) Extraoral photographs at 2‐month and 6‐month follow‐up.

The patient also reported high satisfaction with treatment. At the 3‐year follow‐up, the clinical outcomes remained stable, with no recurrence of symptoms or adverse effects related to PBM.

## 3. Discussion

The present study aimed to describe the clinical outcomes of PBM in two patients with BP, one with chronic facial paralysis and another treated during the acute phase, while also addressing the concomitant management of associated myofascial orofacial pain. The main findings demonstrated that PBM was associated with progressive recovery of facial motor function in both patients, complete or substantial resolution of concomitant myofascial pain, and maintenance of the clinical improvements throughout the follow‐up. Retrospective assessment using the House–Brackmann Facial Nerve Grading System corroborated these clinical findings. Both patients were initially classified as Grade IV (moderately severe dysfunction), indicating marked facial weakness with incomplete eye closure and obvious asymmetry. Following treatment, the chronic case improved to Grade II (mild dysfunction), whereas the acute case achieved Grade I (normal facial function), demonstrating objective improvement in facial motor performance that was consistent with the clinical photographs and functional recovery observed during follow‐up.

These cases highlight that two neuroanatomically distinct clinical entities were managed simultaneously: BP, a motor disorder resulting from dysfunction of the facial nerve (cranial nerve VII), and concomitant orofacial pain, a sensory condition more likely related to trigeminal pathway involvement, myofascial dysfunction, or compensatory muscle overactivity. This distinction is clinically relevant because each condition has different pathophysiological mechanisms and therapeutic targets, although both may benefit from PBM through different biological mechanisms [8, 9].

Although the etiology of BP remains uncertain, several therapeutic approaches have been proposed, including pharmacotherapy, physiotherapy, electrical stimulation, kinesitherapy, and acupuncture [10]. More recently, other adjunctive approaches, such as hyperbaric oxygen therapy, have also demonstrated favorable clinical outcomes when combined with conventional treatment, reinforcing the potential benefits of multidisciplinary management of BP [14]. Among these therapeutic options, PBM has gained increasing attention because of its anti‐inflammatory, analgesic, and neuroregenerative properties, contributing to peripheral nerve repair through modulation of inflammation, enhancement of cellular metabolism, and stimulation of neural regeneration [15].

In Brazil, there are several manufacturers of low‐level laser therapy (LLLT) devices. In this study, two different devices were used based on availability and treatment location: the MMOptics Laser Duo was employed in the chronic case treated in Ecuador, and the DMC Therapy EC device was used in the acute case treated in Brazil. Both are solid‐state GaAlAs diode lasers developed and validated in Brazil. Because of equipment availability, cluster probes, which allow irradiation of broader tissue areas, were not used. Instead, treatment was performed with small spot sizes (0.030 cm^2^ and 0.0984 cm^2^, respectively), enabling precise application to specific anatomical points and trigger areas, particularly important in the facial region. The 808 nm wavelength was selected because of its favorable tissue penetration and its well‐established neuroregenerative, anti‐inflammatory, and analgesic effects [11].

Although identical PBM dosimetric parameters (808 nm, 100 mW, 3 J per point) were used throughout treatment, the biological targets differed according to the irradiated tissues. Irradiation over the affected hemiface was intended to promote facial nerve regeneration, reduce neural inflammation, and facilitate recovery of motor function. Conversely, irradiation of painful muscular trigger points was directed toward controlling myofascial pain through modulation of inflammatory mediators, improvement of local microcirculation, reduction of muscle hyperactivity, and decreased nociceptor sensitization. Therefore, the same irradiation protocol was intentionally maintained because PBM exerts tissue‐dependent biological effects, and the therapeutic objective is determined primarily by the target tissue rather than by different dosimetric parameters. The decision to maintain constant dosimetric parameters while progressively reducing the number of irradiation points reflected the patients′ clinical evolution. As pain resolved and facial motor function improved, treatment became increasingly focused on the remaining functional deficits. This individualized adjustment allowed PBM to be tailored to the changing therapeutic needs throughout rehabilitation.

The chronic case demonstrated that clinically meaningful recovery may still be achieved even when PBM is initiated 4 years after symptom onset. Progressive improvements were observed in facial symmetry, muscle tone, facial expressions, and mouth movements, while pain reduction occurred during the initial treatment sessions. This temporal difference suggests that analgesia was primarily related to modulation of myofascial dysfunction, whereas motor recovery reflected the slower process of peripheral nerve regeneration. The patient had previously undergone pharmacological treatment, electrotherapy, and acupuncture without meaningful improvement and had accepted her condition as permanent. The favorable response to PBM substantially improved her quality of life, particularly regarding facial expression, social interaction, and daily activities. These observations are consistent with previous reports demonstrating that greater facial paralysis severity is associated with poorer quality of life and increased psychosocial burden [9, 16].

Persistent facial dysfunction may also contribute to chronic orofacial pain through compensatory muscle overactivity and the development of myofascial trigger points [17]. Such sequelae have been reported in approximately 5% of BP cases [18]. In the present chronic case, bilateral pain involving masticatory and cervical muscles was likely associated with prolonged facial asymmetry, compensatory muscular activity, and parafunctional habits, explaining the rapid analgesic response following PBM.

Comparison of the two cases demonstrated that PBM was associated with favorable outcomes regardless of the time elapsed since symptom onset. The adolescent patient, treated during the acute phase, exhibited rapid recovery, likely reflecting greater neural plasticity combined with early intervention. In contrast, the adult patient with 4 years of paralysis also experienced clinically significant functional improvement despite previous therapeutic failures, although complete facial symmetry was not achieved. Importantly, both patients received multidisciplinary management, including physiotherapy, pharmacotherapy, and auriculotherapy, emphasizing that PBM should be considered as part of a comprehensive rehabilitation strategy rather than an isolated intervention.

The biological effects of PBM are mainly attributed to stimulation of cytochrome c oxidase within mitochondria, leading to enhanced ATP production, improved cellular metabolism, and tissue repair [19, 20]. PBM has also been shown to modulate oxidative stress, reduce pro‐inflammatory cytokines, and stimulate the release of neurotrophic factors, thereby supporting both neural regeneration and pain control [21]. These biological mechanisms help explain why identical irradiation parameters produced different but complementary therapeutic effects depending on the irradiated tissue.

The favorable outcomes observed in this report are consistent with previous studies supporting PBM as a safe, noninvasive adjunctive therapy capable of promoting neural regeneration while reducing pain and inflammation [12, 19]. No adverse events or complications were observed during treatment or follow‐up, reinforcing its favorable safety profile.

Nevertheless, these findings should not be interpreted as indicating that BP is inherently a painful disorder. Rather, the present cases demonstrate the simultaneous treatment of facial nerve dysfunction and concomitant myofascial pain, two clinically related but neuroanatomically distinct conditions that require separate interpretation of therapeutic outcomes.

Despite the positive clinical outcomes, this report has limitations inherent to the case report design. The absence of a control group, the small number of patients, and the reliance on clinical observation and patient‐reported outcomes limit the generalizability of the findings. In addition, both patients received concomitant therapies, including pharmacological treatment, physiotherapy, and, in the acute case, auriculotherapy. Therefore, the present study design does not allow the independent therapeutic effect of PBM to be isolated from the potential synergistic effects of these interventions. Although photographic documentation supported clinical evaluation, standardized functional scales, and objective electrophysiological assessments, such as electromyography, were not performed. Future randomized controlled clinical trials with larger samples, blinded evaluators, and standardized PBM protocols are necessary to determine the independent contribution of PBM to facial nerve recovery and pain management and to establish evidence‐based treatment recommendations.

### 3.1. Patient Perspective

Both patients reported a high level of satisfaction with the treatment outcomes. The first patient, who had been living with chronic facial paralysis for 4 years, described a significant improvement in her quality of life following PBM therapy. She emphasized the relief of persistent facial pain and the gradual recovery of facial movements, which allowed her to smile more naturally and engage socially with greater confidence. She also highlighted that, after several unsuccessful treatments, PBM provided noticeable benefits that positively impacted her emotional well‐being and daily functioning.

The second patient reported a rapid improvement in symptoms, particularly in pain reduction and facial mobility. She expressed that the recovery of facial symmetry and functional movements restored her self‐esteem and sense of normality. The patient also noted that the treatment was comfortable and easy to tolerate, with no adverse effects experienced throughout the therapy or during long‐term follow‐up. Overall, both patients expressed gratitude for the treatment and perceived PBM as an effective and meaningful component of their care.

## 4. Conclusions

These case reports suggest that PBM may represent a promising adjunctive therapy for both long‐standing and recently diagnosed cases of BP. Although limited by the inherent constraints of case report methodology, the observed clinical improvements in pain control and facial motor function highlight the potential of PBM and support the need for further controlled studies.

PBM also appears to enhance patients′ quality of life and self‐esteem, indicating its value as part of a multidisciplinary treatment approach.

## Author Contributions

Ángel Terrero‐Pérez, María Cecilia Aveiga‐Zambrano, and Mariela Peralta‐Mamani: conceptualization, methodology, and supervision. Renato Yassutaka Faria Yaedú and Gilmar Hugo Paredes‐Muñoz: project administration, validation, and investigation. Omar Andrés Zamorano Castro, Luis Fernández Cotera, Williams Ronald Olivera‐Acuña, and Debora Brasilina Duarte: writing original draft and editing, investigation, and visualization.

## Funding

This study is supported by the Coordenação de Aperfeiçoamento de Pessoal de Nível Superior, 10.13039/501100002322, 88887.967267/2024‐00.

## Disclosure

All authors have read and approved the final version of the manuscript. The corresponding author had full access to all of the data in this study and takes complete responsibility for the integrity of the data and the accuracy of the data analysis.

## Ethics Statement

This manuscript reports a single anonymized case. Written informed consent was obtained from the patients for publication of the case details and accompanying clinical images.

## Consent

The authors certify that the patients involved have given their permission for publication in the journal.

## Conflicts of Interest

The authors declare no conflicts of interest.

## Data Availability

The authors confirm that the data supporting the findings of this study are available within the article.
